# Chemotherapy Decision-Making and Survival Outcomes in Older Women With Early Triple-Negative Breast Cancer: Evidence From Real-World Practice

**DOI:** 10.3389/fonc.2022.867583

**Published:** 2022-04-28

**Authors:** Meng Xiu, Pin Zhang, Qing Li, Peng Yuan, Jiayu Wang, Yang Luo, Fei Ma, Ruigang Cai, Ying Fan, Qiao Li, Binghe Xu

**Affiliations:** Department of Medical Oncology, National Cancer Center/National Clinical Research Center for Cancer/Cancer Hospital, Chinese Academy of Medical Sciences and Peking Union Medical College, Beijing, China

**Keywords:** older women, triple-negative breast cancer, chemotherapy, recurrence, survival

## Abstract

Data regarding chemotherapy options and benefits in older women with early triple-negative breast cancer (TNBC) are limited. Our study aimed to assess the effects of adjuvant chemotherapy on recurrence-free survival (RFS), breast cancer-specific survival (BCSS), and overall survival (OS) rates in elderly TNBC patients. Patients aged ≥65 years diagnosed with stage I-III TNBC (except T1aN0) between 2010 and 2016 were retrospectively included. Multivariate Cox regression was performed to minimize bias. A total of 177 patients were included with a median age of 69 years (range, 65-86), almost all had a Charlson Comorbidity Index of 0-2, and 127 (71.8%) received chemotherapy. Patients who received chemotherapy were younger, had more advanced-stage disease and had better ECOG performance status (*P*<0.05). Among the 127 patients who were administered chemotherapy, 45 (35%) received taxane plus carboplatin, 36 (28%) received anthracycline-and-taxane-based regimens, and 23 (18%) received taxane-based regimens. The regimen options differed based on patient age and tumour stage (*P*<0.05). Nearly 80% of the patients completed ≥6 cycles of chemotherapy, and half had their dosage decreased. After adjustment for confounding factors, patients who received ≥6 cycles of chemotherapy were found to have improved RFS rates (hazard ratio [HR], 0.28; 95% confidence interval [CI], 0.09-0.87; *P*=0.027), and receipt of chemotherapy (≥1 cycle) was associated with better BCSS (HR, 0.19; 95% CI, 0.04-0.97; *P*=0.046) and OS (HR, 0.26; 95% CI, 0.08-0.87; *P*=0.029) rates. These results support the considering the risk for recurrence and performing individualized assessments when determining the appropriate chemotherapy approach for older women with early TNBC.

## Introduction

Despite major improvements in breast cancer survival rates in recent years, such improvements have occurred at a slower rate among older patients than in younger patients (1–3). Triple-negative breast cancer (TNBC), a phenotypic subtype characterized by a lack of oestrogen receptor (ER), progesterone receptor (PR), and human epidermal growth factor receptor 2 (HER2) expression, is an aggressive form of breast cancer associated with a poor prognosis (4, 5). Adjuvant chemotherapy remains the only choice of systemic therapy for early TNBC that reduces the risk for recurrence. The National Comprehensive Cancer Network guidelines indicate that the data are too limited to make adjuvant chemotherapy recommendations for older women with TNBC (6). In clinical practice, chemotherapy for older patients is sometimes difficult to achieve because of declining life expectancy and physiologic status, a greater number of comorbidities, increased toxicity risks, and patient preferences (7, 8).

No large, randomized studies on the value of chemotherapy in older women with early TNBC have been conducted. Furthermore, older patients are underrepresented in available clinical trials due to strict eligibility criteria; thus, making an evidence-based chemotherapy decision for the elderly is challenging (1, 9). Previous retrospective studies have demonstrated conflicting chemotherapy treatment outcomes among elderly TNBC patients. Some studies suggested that advanced age is associated with an indolent disease course and a diminished need for aggressive therapy (10, 11), while others indicated that inadequate treatment contributes to a survival disadvantage (12, 13). Fortunately, two population-based studies (14, 15) have recently been published that analysed data from the National Cancer Database (NCDB) in the USA and the Swedish National Breast Cancer Register (NBCR) and suggested that chemotherapy yielded survival benefits in older women with early TNBC. However, the two national databases did not specify the chemotherapy regimens, dosage, number of cycles, or tolerance levels. These databases also did not provide data on breast cancer recurrence. In the current study, we provide detailed information on the chemotherapy administered and evaluate the benefits of current chemotherapy options in older women with early TNBC in the Chinese population, specifically the effect of chemotherapy on recurrence-free survival (RFS), breast cancer-specific survival (BCSS), and overall survival (OS) rates.

## Methods

### Patients

We retrospectively screened 15,614 consecutive inpatients who were diagnosed with breast cancer and received treatment between January 1, 2010, and December 31, 2016. The eligibility criteria were as follows: 1) female patients aged ≥65 years at diagnosis; 2) patients with pathologically confirmed invasive breast cancer; 3) patients with stage I-III disease; and 4) those with triple-negative breast cancer (ER and PR were defined as negative when <10% of tumour cells showed nuclear staining by immunohistochemistry (IHC); HER2 was considered negative with IHC staining of 0/1+, or fluorescence *in situ* hybridization (FISH) testing confirmed no amplification of the HER2 gene if IHC 2+). Patients were excluded when any of the following conditions were present: 1) recurrent or metastatic breast cancer; 2) noninvasive breast cancer; 3) bilateral breast cancer; 4) other active malignancies; 5) no surgical intervention; 6) T1aN0M0 disease; 7) incomplete ER, PR, and HER2 status; or 8) no survival follow-up information.

### Characteristics

Data including demographic information, medical history, tumour biology, and treatment records were collected from the clinical portal system. Additional treatment information was obtained by physician-directed correspondence. The comorbidities of interest were classified as hypertension, heart disease, cerebrovascular disease, diabetes, chronic respiratory disease, liver disease, chronic renal disease, chronic gastrointestinal disease, connective tissue disease, paralysis and previous history of a different type of cancer. These comorbidities were quantified per patient. The Charlson Comorbidity Index (CCI) was retrospectively evaluated.

### Chemotherapy

Chemotherapy regimens, standard and actual dosages, number of cycles, and tolerance levels were recorded in detail. Patients were considered to have received chemotherapy if they completed at least one cycle of treatment. Patients who received actual doses less than 85% of the recommended in any cycle were considered dosage-decreased, and those who did not complete all the prescribed cycles were considered discontinued.

### Survival

The enrolled patients were closely followed until the cut-off date (December 31, 2019). The primary endpoint was recurrence-free survival (RFS), which was defined as the time from primary treatment (surgery or preoperative chemotherapy) to the time of the first documented invasive breast cancer recurrence event (local, distant, or combined, and contralateral invasive breast cancer) or the date of the last follow-up. Patients who died before experiencing a recurrence event were considered censored at the date of death. Breast cancer-specific survival (BCSS) and overall survival (OS) rates were calculated from the date of primary treatment to the date of death from breast cancer and any cause or the date of the last follow-up.

### Statistics

Patients were divided into two cohorts according to whether they received chemotherapy, and the characteristics between the two cohorts were compared by the chi-square test or Fisher’s exact test, if appropriate. The influence of chemotherapy and other single factors on survival outcomes (RFS, BCSS and OS rates) was estimated by the Kaplan–Meier method. Multivariate Cox proportional hazards models were used to evaluate chemotherapy effects adjusted for other confounding factors. Factors with univariate log-rank *P*<0.2 were included in the multivariate models. In addition, adjustment was made for clinically relevant factors. The Kaplan–Meier method was used to show the effect of chemotherapy on the RFS rate stratified by stage in a *post hoc* analysis. *P* values less than 0.05 were considered statistically significant, and all tests were 2-sided. Statistical analyses were carried out using IBM SPSS Statistics, version 25.

## Results

### Patients and Characteristics

**Figure 1:**
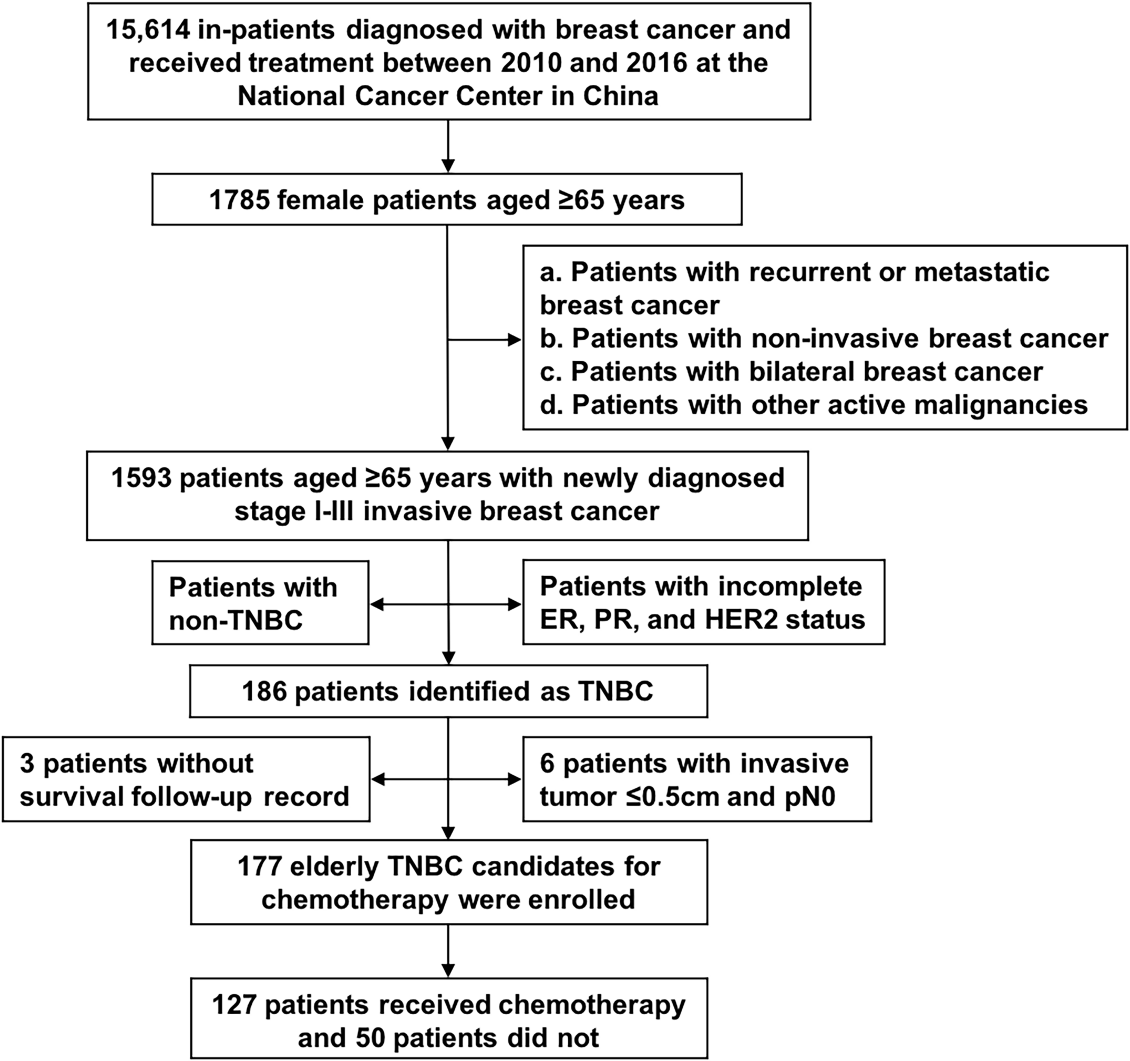
Flowchart of patient selection. ER, estrogen receptor; HER2, human epidermal growth factor receptor 2; PR, progesterone receptor; TNBC, triple-negative breast cancer.

Between January 1, 2010, and December 31, 2016, 15,614 inpatients who were diagnosed with breast cancer and received treatment were screened. A total of 177 older women with early TNBC were included; 127 (71.8%) patients received chemotherapy, while 50 (28.2%) did not (**Figure 1**). The characteristics of the 177 patients are presented in **Table 1**. The median age of the cohort was 69 years (range, 65-86 years). The ECOG performance status values ranged from 0–2, and only 2 patients fit the criteria for ECOG 2. Nearly 80% of the patients had at least one comorbidity, and the most common comorbidities were hypertension (53.7%), heart disease (27.1%), and diabetes (23.2%). Almost all (97.7%) patients had a CCI of 0–2, more than 60% scored 0, and only 4 patients scored ≥3. All the patients underwent surgery, and 33.9% received adjuvant radiation.

**Table 1:** Characteristics of patients.

	All	CT	No CT	*P* ^*^
	N=177 (%)^a^	n=127 (%)^a^	n=50 (%)^a^	
Age				<0.001
65-69	95 (53.7)	83 (65.4)	12 (24.0)	
70-74	42 (23.7)	30 (23.6)	12 (24.0)	
≥75	40 (22.6)	14 (11.0)	26 (52.0)	
Histology				0.107
Ductal	159 (89.8)	117 (92.1)	42 (84.0)	
Other	18 (10.2)	10 (7.9)	8 (16.0)	
Tumor classification^b^				0.975
T1	87 (49.4)	62 (49.2)	25 (50.0)	
T2	81 (46.0)	58 (46.0)	23 (46.0)	
T3,4	8 (4.5)	6 (4.8)	2 (4.0)	
Lymph node status				0.003
N0	108 (61.0)	68 (53.5)	40 (80.0)	
N1	34 (19.2)	27 (21.3)	7 (14.0)	
N2,3	35 (19.8)	32 (25.2)	3 (6.0)	
Stage				0.007
I	63 (35.6)	41 (32.3)	22 (44.0)	
II	76 (42.9)	51 (40.2)	25 (50.0)	
III	38 (21.5)	35 (27.6)	3 (6.0)	
Histological grade				0.308
1,2	64 (36.2)	44 (34.6)	20 (40.0)	
3	96 (54.2)	73 (57.5)	23 (46.0)	
Unknown^c^	17 (9.6)	10 (7.9)	7 (14.0)	
Lymphovascular invasion				0.269
Negative	151 (85.3)	106 (83.5)	45 (90.0)	
Positive	26 (14.7)	21 (16.5)	5 (10.0)	
Ki-67 index				0.732
≤20%	44 (24.9)	33 (26.0)	11 (22.0)	
21%-50%	76 (42.9)	55 (43.3)	21 (42.0)	
>50%	53 (29.9)	36 (28.3)	17 (34.0)	
Unknown^c^	4 (2.3)	3 (2.4)	1 (2.0)	
Hormone receptor				0.353
<1%	152 (85.9)	111 (87.4)	41 (82.0)	
1-9%	25 (14.1)	16 (12.6)	9 (18.0)	
ECOG				<0.001
0	58 (32.8)	53 (41.7)	5 (10.0)	
1-2	119 (67.2)	74 (58.3)	45 (90.0)	
Comorbidity				0.724
No	36 (20.3)	24 (18.9)	12 (24.0)	
1-2 kinds	117 (66.1)	86 (67.7)	31 (62.0)	
≥3 kinds	24 (13.6)	17 (13.4)	7 (14.0)	
Charlson comorbidity index				0.496
0	109 (61.6)	81 (63.8)	28 (56.0)	
1	44 (24.9)	31 (24.4)	13 (26.0)	
≥2	24 (13.6)	15 (11.8)	9 (18.0)	
Body mass index				0.738
<24	70 (39.5)	48 (37.8)	22 (44.0)	
≥24, <28	74 (41.8)	55 (43.3)	19 (38.0)	
≥28	33 (18.6)	24 (18.9)	9 (18.0)	
Surgery^b^				0.161
Mastectomy	145 (82.4)	107 (84.9)	38 (76.0)	
Lumpectomy	31 (17.6)	19 (15.1)	12 (24.0)	
Radiation				<0.001
No	116 (65.5)	72 (56.7)	44 (88.0)	
Yes	60 (33.9)	54 (42.5)	6 (12.0)	
Unknown^c^	1 (0.6)	1 (0.8)	0 (0.0)	

CT, chemotherapy; LVI, lymphovascular invasion.

*The P value was based on Pearson chi-square.

a Some of the percentages did not total 100% due to a rounding error.

b A patient was diagnosed with occult breast cancer and had axillary lymph node dissection.

c We treated unknown data as censored when performing chi-square analysis.

Compared with patients who did not receive chemotherapy, patients who received chemotherapy were younger (*P*<0.001), had more advanced disease (*P*=0.007) [in particular, a higher lymph node burden (*P*=0.003)], had better ECOG performance status (*P*<0.001), and were more likely to receive radiation therapy (*P*<0.001). Of the 50 patients who did not receive chemotherapy, 76% were aged 70 and older, 80% had lymph node-negative disease, only 6.0% had disease in stage III, 90% had an ECOG performance status of 1–2, and 18% had a CCI of ≥2. No significant difference was found in comorbidities or CCI values between patients who received chemotherapy and those who did not (**Table 1**).

### Chemotherapy

**Figure 2:**
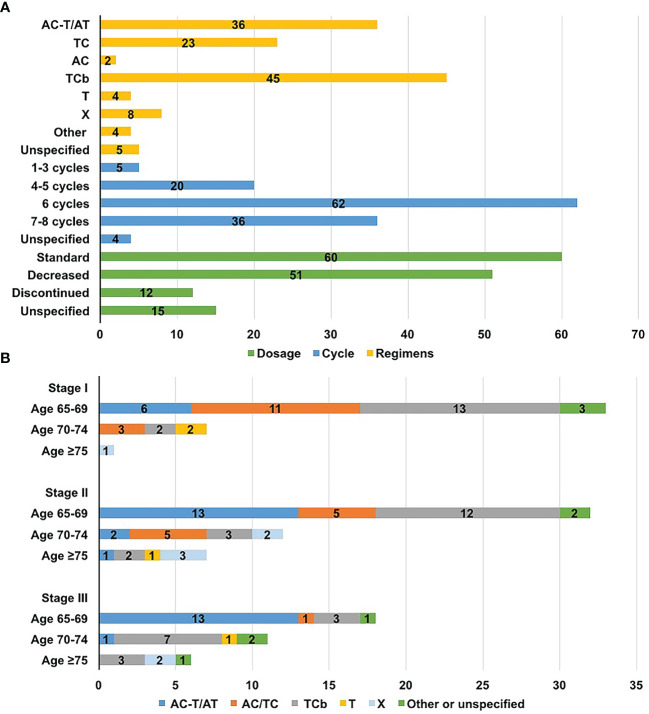
Chemotherapy distributions for older women with triple-negative breast cancer. **(A)** Distribution of regimens, dosage, and the number of cycles; **(B)** Distribution of regimens according to age and stage. A, anthracyclines, including epirubicin or pirarubicin; C, cyclophosphamide; Cb, carboplatin; T, taxane, including docetaxel or paclitaxel; X, capecitabine.

A total of 167 patients were diagnosed with stage T1cN0M0 cancer and higher, 123 of whom received chemotherapy. The remaining 10 patients had T1bN0M0 tumours, and 4 received chemotherapy. Of the 127 patients who received chemotherapy, 116 received adjuvant chemotherapy and 11 received neoadjuvant chemotherapy. A total of 45 (35.4%) patients were treated with taxane plus carboplatin (TCb) as part of a randomized phase II trial (16). Regarding standard chemotherapy, 36 (28.3%) patients received anthracycline-and-taxane-based regimens (AC-T or AT), 23 (18.1%) received taxane-based regimens (TC), and only 2 (1.6%) received anthracycline-based regimens (AC). In addition, 12 (9.4%) patients received taxane or capecitabine monotherapy (**Figure 2A**).

Factors affecting chemotherapy regimens choice are analyzed in **Table 2**. The available regimen options differed based on patient age (*P*=0.023) and disease stage (*P*=0.007) [in particular, the lymph node status (*P*<0.001)]. Anthracycline-and-taxane-based regimens were primarily administered to patients aged 65-69 years and those in stage II to III. Patients aged ≥70 were more likely to receive carboplatin-based chemotherapy instead of anthracycline-containing regimens, with more than half of them receiving TCb. Patients treated with anthracycline-or-taxane-based regimens typically had earlier stage disease, and only 2 had positive nodes (**Figure 2B** and **Table 2**).

**Table 2:** Major chemotherapy regimens according to clinical factors.

	AC-T/AT	AC/TC	TCb	*P* ^*^
Age, n (%)				0.023
65-69	32 (41.6)	17 (22.1)	28 (36.4)	
≥70	4 (13.8)	8 (27.6)	17 (58.6)	
Tumor classification, n (%)				0.837
≤2cm	16 (31.4)	13 (25.5)	22 (43.1)	
>2cm	20 (36.4)	12 (21.8)	23 (41.8)	
Lymph node status, n (%)				<0.001
Negative	10 (16.9)	23 (39.0)	26 (44.1)	
Positive	26 (55.3)	2 (4.3)	19 (40.4)	
Stage, n (%)				0.007
I	6 (17.1)	14 (40.0)	15 (42.9)	
II	16 (37.2)	10 (23.3)	17 (39.5)	
III	14 (50.0)	1 (3.6)	13 (46.4)	
Histological grade, n (%) ^#^				0.944
1,2	11 (30.6)	9 (25.0)	16 (44.4)	
3	21 (33.9)	15 (24.2)	26 (41.9)	
Lymphovascular invasion, n (%)				0.295
Negative	30 (32.6)	24 (26.1)	38 (41.3)	
Positive	6 (42.9)	1 (7.1)	7 (50.0)	
Ki-67 index, n (%)^#^				0.323
≤20%	9 (32.1)	4 (14.3)	15 (53.6)	
>20%	27 (36.0)	19 (25.3)	29 (38.7)	
Hormone receptor, n (%)				0.467
<1%	29 (32.2)	23 (25.6)	38 (42.2)	
1-9%	7 (43.8)	2 (12.5)	7 (43.8)	
ECOG, n (%)				0.926
0	16 (34.8)	10 (21.7)	20 (43.5)	
1-2	20 (33.3)	15 (25.0)	25 (41.7)	

A, anthracyclines, including epirubicin or pirarubicin; C, cyclophosphamide; Cb, carboplatin; T, taxane, including docetaxel or paclitaxel.

*The P value was based on Pearson chi-square.

^#^We treated unknown data as censored.

The median number of cycles of chemotherapy was 6 (range, 1-8 cycles), and 77.2% of the patients completed at least 6 cycles of chemotherapy. A total of 12 (9.4%) patients discontinued prescribed regimens due to poor tolerance (gastrointestinal reaction, 3; myelosuppresion, 5; fatigue 3; unknown, 1). Of the 112 patients with detailed drug dosage records, the dosage was decreased in 51 (45.5%), and 31 began chemotherapy with reduced dosages. The main reason for the dosage reductions was neutropenia. Of the 37 patients who received paclitaxel, 5 (13.5%) were dosage-reduced due to neurotoxicity. And among 61 patients receiving docetaxel or paclitaxel liposome, no serious neurotoxicity was documented (**Figure 2A**).

### Survival

Within a median follow-up period of 59 months (range, 5–118 months), the 5-year RFS, BCSS, and OS rates of the 177 patients were estimated to be 82.9% (95% confidence interval [CI] 77.0-88.8%), 92.7% (95% CI, 88.4-97.0%), and 88.3% (95% CI, 83.0-93.6%), respectively. Univariate associations between clinical factors and outcomes are shown in **Table 3**. The 5-year estimated RFS rate was 83.3% (95% CI, 76.6-90.0%) among patients treated with chemotherapy versus 81.7% (95% CI, 69.9-93.5%) among patients who did not receive chemotherapy (log-rank *P*=0.819). A similar result was observed for the 5-year BCSS rate (93.7% [95% CI, 89.0-98.4] vs. 90.0% [95% CI, 80.4-99.6%]; log-rank *P*=0.884). The 5-year OS rate was 91.1% among patients receiving chemotherapy (95% CI, 85.8-96.4%) and 80.7% (95% CI, 68.2-93.2%) among those who did not receive chemotherapy (log-rank *P*=0.385).

**Table 3:** Univariate analysis of association between clinical factors and 5-year survival outcomes in 177patients.

	N	Recurrence-free Survival			Breast Cancer-specific Survival			Overall Survival		
		n^a^	5-y Estimated, % (95% CI)	P^*^	n^b^	5-y Estimated, % (95% CI)	P^*^	n^c^	5-y Estimated, % (95% CI)	P^*^
All	177	31	82.9 (77.0-88.8)		16	92.7 (88.4-97.0)		23	88.3 (83.0-93.6)	
Chemotherapy				0.819			0.884			0.385
No	50	8	81.7 (69.9-93.5)		4	90.0 (80.4-99.6)		8	80.7 (68.2-93.2)	
Yes	127	23	83.3 (76.6-90.0)		12	93.7 (89.0-98.4)		15	91.1 (85.8-96.4)	
Cycles^d^				0.941			0.147			0.099
0	50	8	81.7 (69.9-93.5)		4	90.0 (80.4-99.6)		8	80.7 (68.2-93.2)	
1-5	25	4	83.0 (67.7-98.3)		0	100		0	100	
≥6	98	18	82.6 (74.8-90.4)		12	91.7 (85.6-97.8))		15	88.4 (81.3-95.5)	
Age				0.028			0.192			0.102
65-69	95	13	88.0 (81.3-94.7)		7	94.5 (89.8-99.2)		8	93.5 (88.4-98.6)	
70-74	42	13	65.8 (50.3-81.3)		7	86.4 (75.0-97.8)		8	84.0 (72.0-96.0)	
≥75	40	5	88.5 (77.7-99.3)		2	95.2 (86.2-100.0)		7	79.8 (64.5-95.1)	
Stage				<0.001			<0.001			<0.001
I	63	3	94.7 (88.8-100.0)		0	100.0		1	98.4 (95.3-100.0)	
II	76	11	86.7 (78.5-94.9)		5	94.9 (89.0-100.0)		10	87.0 (78.4-95.6)	
III	38	17	55.8 (39.3-72.3)		11	76.0 (61.3-90.7)		12	74.0 (59.1-88.9)	
Grade^d^				0.263			0.941			0.529
1,2	64	8	90.2 (82.8-97.6)		5	94.4 (88.1-100.0)		6	92.8 (85.9-99.7)	
3	96	18	80.4 (71.8-89.0)		7	95.6 (91.3-99.9)		12	89.2 (82.3-96.1)	
LVI				<0.001			<0.001			<0.001
Negative	151	19	88.4 (82.9-93.9)		8	97.6 (94.9-100.0)		14	93.0 (88.5-97.5)	
Positive	26	12	51.9 (31.7-72.1)		8	61.8 (38.9-84.7)		9	58.6 (36.1-81.1)	
Ki-67^d^				0.665			0.831			0.364
≤20%	44	7	90.1 (80.9-99.3)		4	95.5 (89.4-100.0)		4	95.5 (89.4-100.0)	
21%-50%	76	15	79.2 (69.8-88.6)		8	90.2 (82.6-97.8)		12	84.1 (74.9-93.3)	
>50%	53	8	83.5 (72.9-94.1)		4	93.9 (87.2-100.0)		7	88.2 (79.4-97.0)	
HR				0.311			0.667			0.326
<1%	152	28	82.2 (75.7-88.7)		14	93.1 (88.6-97.6)		21	87.8 (82.1-93.5)	
1-9%	25	3	87.8 (74.9-100.0)		2	91.2 (79.4-100.0)		2	91.2 (79.4-100.0)	
ECOG				0.625			0.542			0.122
0	58	9	85.4 (76.0-94.8)		4	93.3 (85.7-100.0)		4	93.3 (85.7-100.0)	
1-2	119	22	81.7 (74.3-89.1)		12	92.4 (87.3-97.5)		19	85.9 (79.2-92.6)	
Comorbidity				0.460			0.587			0.314
No	36	5	84.3 (71.6-97.0)		2	93.0 (83.4-100.0)		3	87.8 (74.5-100.0)	
1-2 kinds	117	20	83.3 (76.2-90.4)		11	93.1 (88.0-98.2)		15	89.8 (83.9-95.7)	
≥3 kinds	24	6	78.3 (61.6-95.0)		3	90.7 (78.4-100.0)		5	81.3 (64.6-98.0)	
CCI				0.685			0.273			0.802
0	109	18	83.6 (76.2-91.0)		8	93.1 (87.6-98.6)		13	88.0 (81.1-94.9)	
1	44	10	79.2 (67.0-91.4)		7	90.8 (82.2-99.4)		7	90.8 (82.2-99.4)	
≥2	24	3	87.3 (73.8-100.0)		1	95.0 (85.4-100.0)		3	84.4 (68.1-100.0)	
BMI				0.496			0.790			0.821
<24	70	10	86.7 (78.7-94.7)		7	93.7 (87.6-99.8)		8	92.0 (85.1-98.9)	
24-27.9	74	13	79.3 (68.9-89.7)		5	91.5 (83.9-99.1)		10	83.7 (73.9-93.5)	
≥28	33	8	81.1 (67.4-94.8)		4	93.3 (84.3-100.0)		5	90.5 (80.3-100.0)	
Radiation^d^				0.176			0.046			0.585
No	116	16	86.7 (80.0-93.4)		6	95.7 (91.6-99.8)		13	88.7 (82.2-95.2)	
Yes	60	14	75.7 (64.5-86.9)		9	87.1 (78.1-96.1)		9	87.1 (78.1-96.1)	

BMI, body mass index; CCI, charlson comorbidity index; CI, confidence interval; HR, hormone receptor; LVI, lymphovascular invasion.

*The P value was tested by log-rank method.

a Invasive breast cancer recurrence event.

b Breast cancer specific death event.

c All cause death event.

d Patients with unknown data were not analyzed.

Recurrence events were recorded in 31 (17.5%) patients; 14 patients experienced the first recurrence at locoregional sites, 12 at distant sites, and 4 in locoregional and distant sites combined. And one patient developed contralateral invasive breast cancer. The median RFS duration of patients with recurrence events was 26 months (range, 4-94 months), and 64.5% of the recurrence events were observed during the first 3 years after diagnosis. During the follow-up period, 16 (9.0%) patients died from breast cancer, 7 (4.0%) patients died from other diseases (the main causes were pneumonia and heart failure), and no treatment-related deaths occurred. The rate of death from other diseases among patients who did not receive chemotherapy was higher (**Table S1**).

**Table 4** shows the results of the multivariate Cox survival analysis. Receipt of chemotherapy (defined as completing at least one cycle) was associated with better a BCSS rate (hazard ratio [HR], 0.19; 95% CI, 0.04-0.97; *P*=0.046) and OS (HR, 0.26; 95% CI, 0.08-0.87; *P*=0.029) after adjusting for age, stage, lymphovascular invasion (LVI), CCI, and radiation but failed to impact the RFS rate (HR, 0.44; 95% CI, 0.16-1.19; *P*=0.104; **Table S2**). Patients who received at least 6 cycles of chemotherapy had improved RFS rates after adjustment compared to those who did not receive chemotherapy (HR, 0.28; 95% CI, 0.09-0.87; *P*=0.027). In addition, stage III disease and the presence of LVI were demonstrated to be independent determinants of poorer RFS, BCSS, and OS outcomes (**Table 4**).

**Table 4:** Multivariate Cox proportional hazard model of factors associated with survival outcomes in 177 patients.

		Recurrence-free Survival		Breast Cancer-specific Survival		Overall Survival	
		HR (95% CI)	*P*	HR (95% CI)	*P*	HR (95% CI)	*P*
Chemotherapy^a^	No	1		1		1	
	1-5 cycles	0.97 (0.27-3.53)	0.962	0.19 (0.04-0.97)^a^	0.046	0.26 (0.08-0.87)^a^	0.029
	≥6 cycles	0.28 (0.09-0.87)	0.027				
Age	65-69	1		1		1	
	70-74	1.87 (0.83-4.23)	0.131	1.62 (0.55-4.77)	0.386	1.65 (0.61-4.45)	0.321
	≥75	0.51 (0.14-1.85)	0.308	0.45 (0.05-4.33)	0.488	1.56 (0.45-5.37)	0.485
Stage	I	1		1^b^		1	
	II	3.66 (0.94-14.17)	0.061			6.99 (0.87-56.19)	0.067
	III	16.34 (3.74-71.42)	<0.001	9.27 (1.97-43.66)	0.005	26.81 (2.82-255.25)	0.004
LVI	Negative	1		1		1	
	Positive	2.49 (1.02-6.08)	0.045	5.11 (1.50-17.37)	0.009	3.43 (1.27-9.26)	0.015
CCI	0-1	1		1		1	
	≥2	0.86 (0.26-2.87)	0.803	0.50 (0.06-3.99)	0.517	1.17 (0.34-4.03)	0.806
Radiation	No	1		1		1	
	Yes	0.87 (0.32-2.36)	0.779	0.92 (0.22-3.85)	0.904	0.81 (0.25-2.59)	0.717

CCI, charlson comorbidity index; CI, confidence interval; HR, hazard ratio; LVI, lymphovascular invasion.

a When analyzing BCSS and OS, chemotherapy was classified as no or yes (including 1-5 cycles and ≥6 cycles).

b The reference group was changed to patients in stage I-II, because no patients in stage I died from breast cancer.

**Figure 3:**
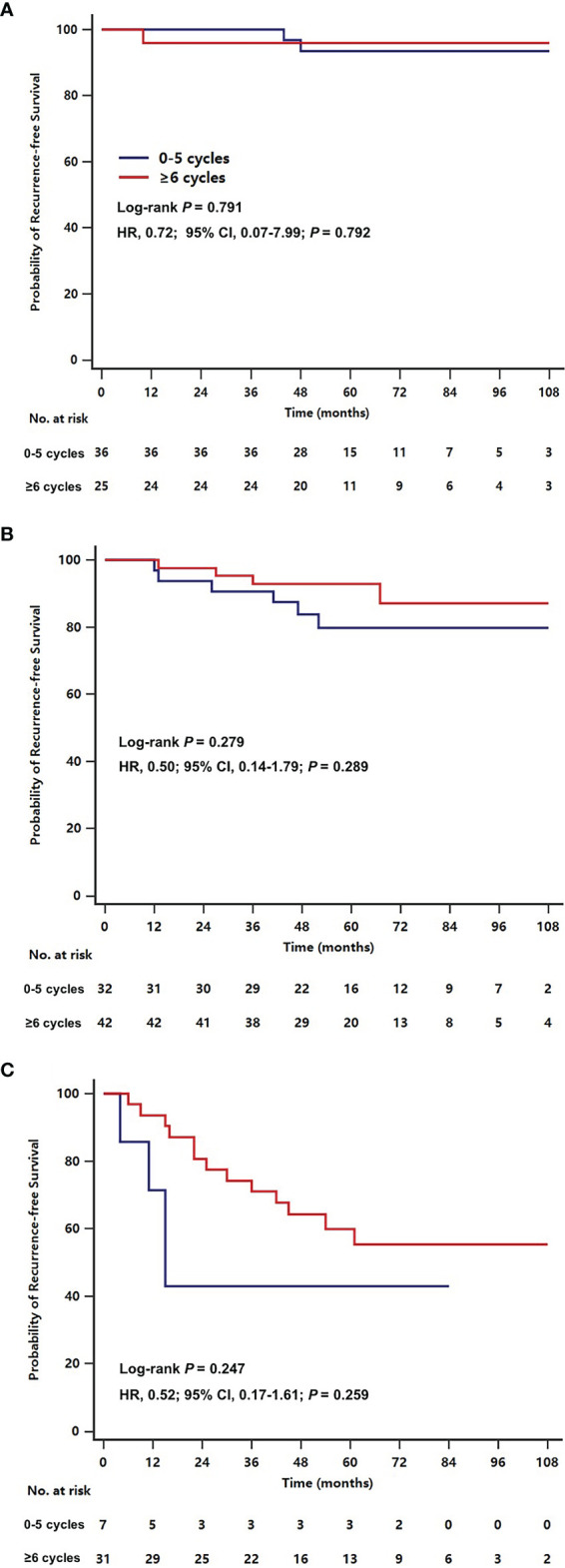
Kaplan-Meier plots for recurrence-free survival by receiving ≥6 or 0-5 cycles of chemotherapy in older women with stage I **(A)**, stage II **(B)**, stage III **(C)** triple-negative breast cancer.

**Figure 3** shows the effect of ≥6 cycles of chemotherapy on the RFS rate stratified by stage in a subgroup analysis. The estimated 5-year RFS rate of patients with stage I disease was 96.0% (95% CI, 88.4-100.0%) among those who received ≥6 cycles of chemotherapy and 93.3% (95% CI, 84.3-100%) among those who received 0-5 cycles of chemotherapy (HR, 0.71; 95% CI, 0.06-7.81; *P*=0.778). The 5-year RFS rate of patients with stage II disease was 92.5% (95% CI, 84.3-100%) among patients who received ≥6 cycles of chemotherapy and 78.5% (95% CI, 63.0-94.0%) among those who received 0-5 cycles (HR, 0.50; 95% CI, 0.14-1.76; *P*=0.278). The 5-year RFS rate of patients with stage III disease was 59.9% (95% CI, 42.1-77.7%) among those who received ≥6 cycles and 34.3% (95% CI, 0.0-72.5%) among those who received 0-5 cycles (HR, 0.39; 95% CI, 0.13-1.21; *P*=0.102).

We further explored the chemotherapy benefit for patients in different subgroups of age and no significant result was found. The HR for the effect of chemotherapy versus no chemotherapy on RFS was 0.93 (95% CI, 0.20-4.23; *P*=0.924) for patients aged <70 years and 1.64 (95% CI, 0.62-4.38; *P*=0.322) for those aged ≥70 (**Figure S1**).

There were no significant differences in RFS outcomes among patients treated with the different regimens (log rank *P*=0.702). The 5-year RFS rates of patients who received taxane plus carboplatin, anthracycline-and-taxane-based regimens, and anthracycline-or-taxane-based regimens were estimated to be 88.0% (95% CI, 78.0-98.0%), 86.0% (95% CI, 74.6-97.4%), and 80.0% (95% CI, 64.3-95.7%), respectively (**Figure S2**).

## Discussion

In the present study of 177 women aged 65 and older with early TNBC at a single institute over a period of 7 years, approximately 70% received chemotherapy. Chemotherapy decision-making and the regimen options varied based on patient age and disease stage. After adjustment for confounding factors, the findings indicated a significant BCSS and OS outcome benefit of chemotherapy (defined as completing at least one cycle). At least 6 cycles of chemotherapy improved the RFS rate in comparison with no chemotherapy in the multivariable analysis. The current study is of particular importance due to the lack of randomized data available to guide adjuvant chemotherapy choices for older women with TNBC.

All older women enrolled in our study were candidates for chemotherapy from the view of clinicopathologic features based on guidelines for the general population (6, 17); however, only a subset of these patients received chemotherapies, and patient age and disease stage (especially lymph node involvement) were demonstrated to be determinants for clinical decision-making, consistent with the results of a population-based study (18) of lymph node (LN)-positive, ER-positive elderly patients. Data from different sources reflect the current status of chemotherapy selection for elderly TNBC patients. In our study, 53.7% of patients aged ≥70 years received chemotherapy. The NCDB-based study in the USA (14) reported that 46.6% of women aged ≥70 with TNBC were treated with chemotherapy, and 16.6% were recommended for chemotherapy but ultimately did not receive it. The NCDB-based study (14) reported that the proportions of patients with N0, N1, and N2-3 disease who received chemotherapy were 40.9%, 60.7%, and 64.4%, respectively. In our cohort, 63.0%, 79.4%, and 91.4% of patients with N0, N1, and N2-3 disease received chemotherapy, respectively. The younger age and better performance status of our cohort might partly explain the higher proportions. Although patients in our study who received chemotherapy had significantly better ECOG performance status than patients who did not, almost all ranged from ECOG 0-1, and they were all eligible for chemotherapy based on tolerance. Chemotherapy decision-making for older cancer patients is far more complex than that for younger patients, and other factors, such as life expectancy, patient preference, financial position, and the presence of comorbidities, must be considered comprehensively (7).

In the current study, comorbidity and the Charlson Comorbidity Index (CCI) failed to affect chemotherapy decisions and survival outcomes. A previous study revealed that chemotherapy is rarely recommended for older breast cancer patients with a CCI value ≥3 (3), and that patients with a higher CCI value had significantly worse OS outcomes (14, 15). Based on the comorbid conditions listed in the CCI, we considered numerous highly prevalent comorbidities, such as hypertension (19, 20). Although nearly 80% of patients in our cohort had comorbidities, few had diseases that severely impacted their functional status, as only 4 patients scored CCI ≥3, which may partly explain the lack of correlation between comorbidities and treatment decisions or survival outcomes. The sample size of our study was smaller than that of other population-based studies (14, 15), which may have limited our ability to analyze survival in relation to CCI values. The findings indicate that individualized assessment using developed tools such as the CCI are beneficial for estimating life expectancy and functional status among older patients with multiple comorbidities. And we are not supposed to prevent the elderly from chemotherapy blindly (7, 21).

Our study provides detailed information about chemotherapy regimens, dosage, number of cycles, and distributions of regimens according to patient age and disease stage among older women with TNBC in a clinical setting, representing an initial attempt to provide a reference for clinical decision-making. Limited randomized data (22–24) show that standard chemotherapy regimens remain recommended for older women with breast cancer, but no study has focused only on elderly patients with TNBC. Of the patients who received chemotherapy in our study, barely 30% received anthracycline-containing regimens. It has been demonstrated that older breast cancer patients can obtain survival benefits from anthracycline, mainly at the cost of increased risks for haematological toxicity and cardiotoxicity compared with those observed in younger patients (25–27). Taxane plus carboplatin (TCb) was the most common regimen used in our study due to the participation of patients in a randomized phase 2 trial (16) comparing 6 cycles of TCb with anthracycline-and-taxane-based regimen (AC-T) as adjuvant chemotherapy for early TNBC; the patients had a median age of 48 in this study, which was conducted between 2009 and 2015. The results of this randomized trial (16) indicated that the effects of different regimens were similar, consistent with the results of our study. In our study, patients aged ≥70 were more likely to receive TCb than AC-T. At the same time, the patients treated with TCb in the randomized trial showed better compliance. Currently, the routine use of platinum agents in the adjuvant setting is not recommended (6, 17). We speculate that TCb may be a feasible regimen for older women with TNBC who cannot tolerate anthracycline-containing regimens, and further investigations are required.

In our analysis, older women with TNBC who received chemotherapy had improved BCSS and OS rates after adjustment. Consistent with the other two studies on chemotherapy for elderly patients with TNBC (14, 15), the multivariate analysis of different cohorts supported considering chemotherapy for older women with TNBC. The different sample sizes might be conducive to the relatively lower HR in our cohort. The median 59-month follow-up in our study was 2 years longer than that in the other two studies. In our univariate analysis, no significant survival outcome differences were found between the patients who received chemotherapy and those who did not, as patients who received chemotherapy had more advanced disease, indicating that oncologists carefully selected patients likely to benefit from chemotherapy.

Our specific follow-up information makes our study the first to evaluate the effect of chemotherapy on RFS among older women with TNBC in the real world. Considering the competing causes of death in the older population (22), we excluded non-breast cancer death when defining RFS events. In our study, receipt of chemotherapy (defined as completing ≥1 cycle) failed to impact RFS outcomes, while ≥6 cycles of chemotherapy reduced the risk for breast cancer recurrence. This result reminds us that adequate dosing is necessary to ensure the effectiveness of therapeutics. However, administration of at least 6 cycles of chemotherapy did not have a significant effect on RFS among patients stratified by disease stage, which may be largely explained by the small sample size of patients at each disease stage. The addition of patients who received 1-5 cycles of chemotherapy to the reference groups could be another cause of this finding, diluting the impact of ≥6 cycles of chemotherapy. The absolute improvements in RFS rates increased as disease stage increased. Although we excluded patients with T1aN0M0 disease, the remaining elderly TNBC patients with stage I disease had a favourable prognosis regardless of whether they received chemotherapy. Future studies are required to determine whether chemotherapy can be avoided in a subset of low-risk elderly TNBC patients who would be unlikely to obtain sufficient chemotherapy benefits to outweigh the negative effects of treatment toxicities.

Our study has limitations inherent to all retrospective studies, rendering it prone to confounding. When interpreting the results of our study, we should be conscious of the heterogeneity in patient characteristics and chemotherapy regimens. Another main weakness of our current study is the single-institute design and limited population size. Just as every coin has two sides, however, the single-institute design ensures the consistency of biology testing and the relative consistency of treatment. Third, the cut-off values of hormone receptor negativity were modified to <1% in the later College of American Pathologists guidelines, and we enrolled patients guided by the old criteria. However, the biology of tumors with HR 1-9% stained had been demonstrated similar to that with HR <1%. Last but not least, the absence of the individualized Comprehensive Geriatric Assessment (CGA) data limited our understanding of the health status of the older patients studied.

In conclusion, we found that chemotherapy is associated with significant RFS, BCSS, and OS benefits among older women with early TNBC. Oncologists carefully selected patients likely to benefit from chemotherapy and administered the appropriate treatment regimens, providing a valuable reference to assist in clinical decision-making in the real world. Prospective clinical trials evaluating chemotherapies for elderly TNBC patients under the guidance of CGA are urgently needed to optimize treatment strategies.

## Data Availability Statement

The original contributions presented in the study are included in the article/**Supplementary Material**. Further inquiries can be directed to the corresponding author.

## Ethics Statement

The studies involving human participants were reviewed and approved by Research and Ethics Committee of the National Cancer Center in China. The patients/participants provided their written informed consent to participate in this study.

## Author Contributions

MX and PZ had full access to all of the data in the study and take responsibility for the integrity of the data and the accuracy of the data analysis. PZ and BX made substantial contributions to the conception and design of the work. All the authors did the acquisition, analysis, or interpretation of data. MX drafted the manuscript and did the critical revision of the manuscript for important intellectual content. All authors contributed to the article and approved the submitted version.

## Funding

This work was supported by the National Key R&D Program of China (2020YFC2004800).

## Conflict of Interest

The authors declare that the research was conducted in the absence of any commercial or financial relationships that could be construed as a potential conflict of interest.

## Publisher’s Note

All claims expressed in this article are solely those of the authors and do not necessarily represent those of their affiliated organizations, or those of the publisher, the editors and the reviewers. Any product that may be evaluated in this article, or claim that may be made by its manufacturer, is not guaranteed or endorsed by the publisher.

## Supplementary Material

Supplementary Figure 1 Kaplan-Meier plots for recurrence-free survival by age and chemotherapy in older women with triple-negative breast cancer.

Supplementary Figure 2 Kaplan-Meier plots for recurrence-free survival by different regimens in older women with triple-negative breast cancer.

## Abbreviations

A, anthracyclines; BCSS, breast cancer-specific survival; BMI, body mass index; C, cyclophosphamide; Cb, carboplatin; CCI, Charlson Comorbidity Index; CGA, Comprehensive Geriatric Assessment; CI, confidence interval; CT, chemotherapy; ER, estrogen receptor; FISH, fluorescence *in situ* hybridization; HER2, human epidermal growth factor receptor 2; HR, hormone receptor; HR, hazard ratio; IHC, immunohistochemistry; LN, lymph node; LVI, lymphovascular invasion; NBCR, National Breast Cancer Register; NCDB, National Cancer Database; OS, overall survival; PR, progesterone receptor; RFS, recurrence-free survival; T, taxane; TNBC, triple-negative breast cancer; X, capecitabine.
