# Supplementary figures and images for: Chemotherapy Decision-Making and Survival Outcomes in Older Women With Early Triple-Negative Breast Cancer: Evidence From Real-World Practice
Source: Front Oncol. 2022 Apr 28;12:867583. doi: 10.3389/fonc.2022.867583 (PMC9097590; doi:10.3389/fonc.2022.867583)

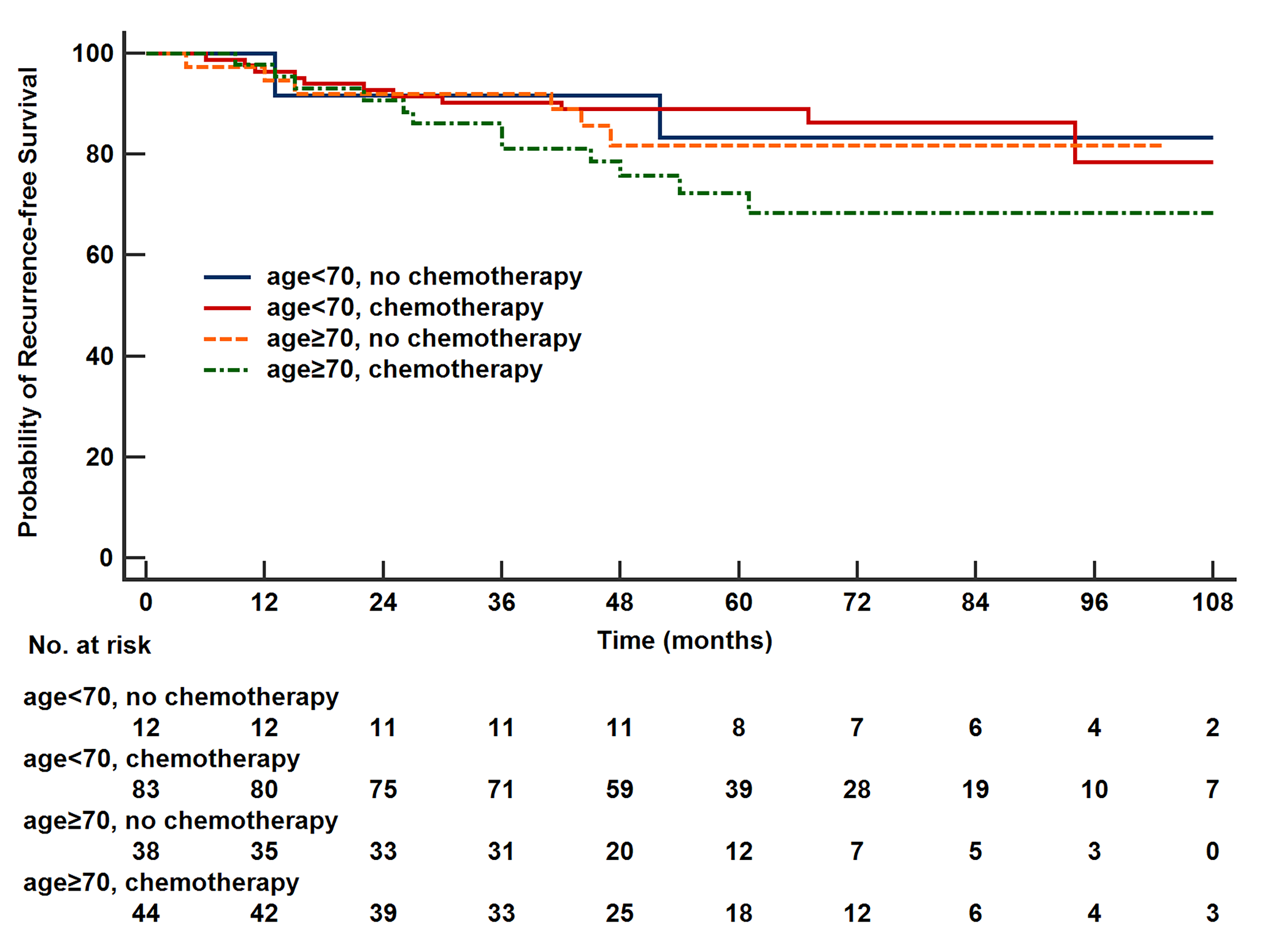

Supplement: Supplementary Figure 1 — Kaplan-Meier plots for recurrence-free survival by age and chemotherapy in older women with triple-negative breast cancer. [file Image_1.tif]

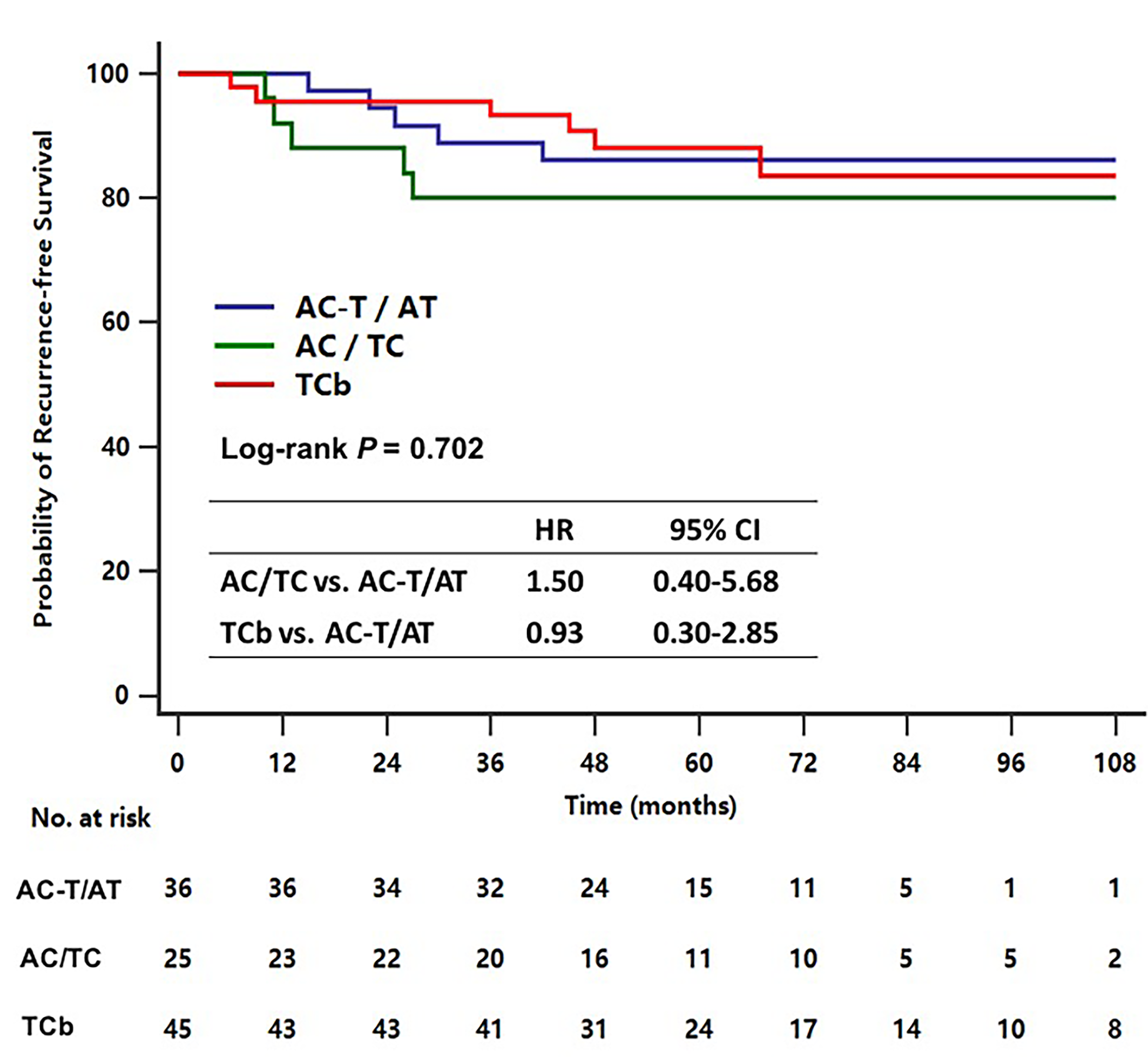

Supplement: Supplementary Figure 2 — Kaplan-Meier plots for recurrence-free survival by different regimens in older women with triple-negative breast cancer. [file Image_2.tif]
