# Supplementary material for: Chemotherapy Decision-Making and Survival Outcomes in Older Women With Early Triple-Negative Breast Cancer: Evidence From Real-World Practice
Source: Front Oncol. 2022 Apr 28;12:867583. doi: 10.3389/fonc.2022.867583 (PMC9097590; doi:10.3389/fonc.2022.867583)
Supplement: Supplementary file 3 [file Table_1.pdf]

Table S1. Events at a median follow-up of 59 months

Event	All<br>N=177 (%)	Chemotherapy<br>n=127 (%)	No Chemotherapy<br>n=50 (%)
**First recurrence**	31 (17.5)	23 (18.1)	8 (16.0)
Locoregional only	14 (7.9)	10 (7.9)	4 (8.0)
Distant only	12 (6.8)	9 (7.1)	3 (6.0)
Combined	4 (2.3)	3 (2.4)	1 (2.0)
Contralateral invasive breast cancer	1 (0.6)	1 (0.8)	0 (0.0)
**Death**	23 (13.0)	15 (11.8)	8 (16.0)
Breast cancer	16 (9.0)	12 (9.4)	4 (8.0)
Other	7 (4.0)	3 (2.4)	4 (8.0)
Pneumonia	2	1	1
Heart failure	2	0	2
Pulmonary embolism	1	1	0
Gastrointestinal bleeding	1	0	1
Vaginal cancer	1	1	0
**Second primary cancer**	8 (4.5)	6 (4.7)	2 (4.0)
Lymphoma	1	0	1
Lung	2	2	0
Esophageal	1	1	0
Pancreatic	1	0	1
Renal	1	1	0
Ovarian	1	1	0
Vaginal	1	1	0
