# Supplementary material for: Chemotherapy Decision-Making and Survival Outcomes in Older Women With Early Triple-Negative Breast Cancer: Evidence From Real-World Practice
Source: Front Oncol. 2022 Apr 28;12:867583. doi: 10.3389/fonc.2022.867583 (PMC9097590; doi:10.3389/fonc.2022.867583)
Supplement: Supplementary file 4 [file Table_2.pdf]

Table S2. Multivariate Cox proportional hazard model of factors associated with recurrence-free survival

		**Recurrence-free Survival**	
		**HR (95% CI)**	***P***
Chemotherapy	No	1	
	Yes	0.42 (0.15-1.13)	0.085
Age	65-69	1	
	70-74	1.75 (0.78-3.94)	0.174
	≥75	0.51 (0.14-1.82)	0.298
Stage	I	1	
	II	2.81 (0.75-10.46)	0.125
	III	12.08 (2.82-51.70)	0.001
LVI	Negative	1	
	Positive	2.14 (0.90-5.09)	0.084
Comorbidity	0-2 kinds	1	
	≥3 kinds	1.51 (0.55-4.09)	0.423
Radiation	No	1	
	Yes	0.74 (0.27-2.00)	0.545

Abbreviations: CI, confidence interval; HR, hazard ratio; LVI, lymphovascular invasion.
